# Supplementary material for: Black carbon scavenging by low-level Arctic clouds
Source: Nat Commun. 2023 Sep 7;14:5488. doi: 10.1038/s41467-023-41221-w (PMC10485071; doi:10.1038/s41467-023-41221-w)
Supplement: Supplementary file 1 — Supplementary Information [file 41467_2023_41221_MOESM1_ESM.pdf]

Supplementary information for “Black carbon scavenging by low-level Arctic clouds”

Paul Zieger[1,2], Dominic Heslin-Rees[1,2], Linn Karlsson[1,2], Makoto Koike[3], Robin Modini[4], and Radovan Krejci[1,2]

[1]Department of Environmental Science, Stockholm University, Stockholm, Sweden
[2]Bolin Centre for Climate Research, Stockholm University, Stockholm, Sweden
[3]Department of Earth and Planetary Science, University of Tokyo, Tokyo, Japan
[4]Laboratory of Atmospheric Chemistry, Paul Scherrer Institute, Villigen, Switzerland

Correspondence: paul.zieger@aces.su.se

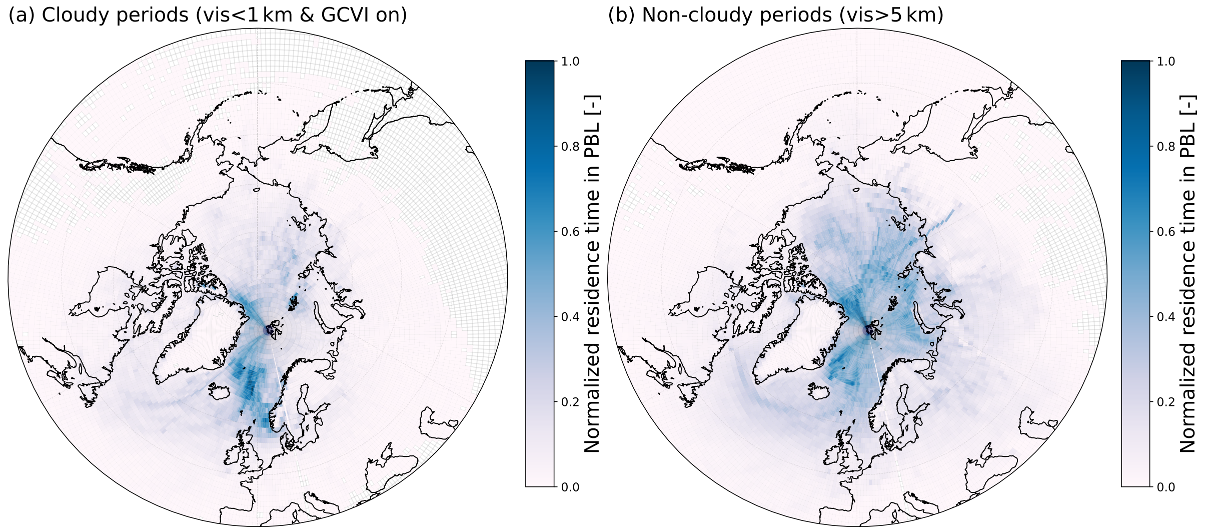

Figure S1. Surface residence time for cloudy and non-cloudy periods for entire 4-year data set. (a) Surface residence time for cloudy periods with GCVI sampling (visibility$<$1 km). (b) Surface residence time for non-cloudy periods (visibility$>$5 km). Grid cells in which no back trajectories traversed over are given black edges. The surface residence times were normalised by dividing by the maximum count found in the grid cells (not including the grid cell in which ZEP is located).

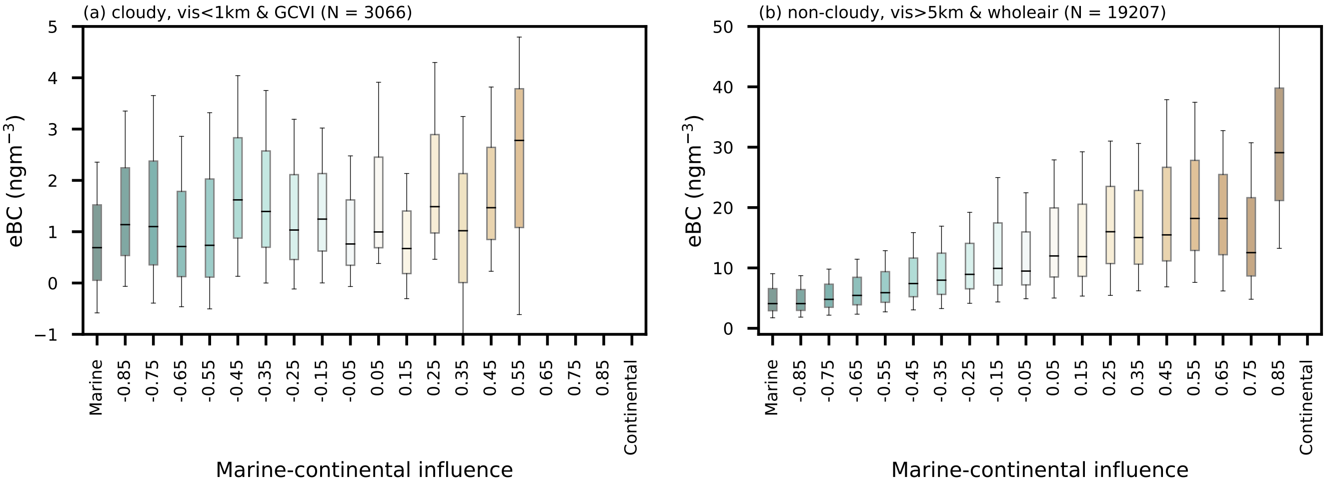

Figure S2. Marine-continental influence. (a) Dependence of eBC on surface type for cloudy periods with GCVI sampling (visibility$<$1 km). (b) Dependence of eBC on surface type for non-cloudy periods (visibility$>$5 km). Here, each endpoint is denoted as being over land (1) or ocean (-1), and the average of all endpoints within the mixed-layer for each ensemble is then assigned to an observation. Hence, an ensemble which traverses only over the ocean is given the value -1. The fill color of the box plots denotes the values of the x-axis. The centre line of the boxes represents the median, while the extent of the boxes show the interquartile range. The whisker show the range of data (defined as 1.5 times the interquartile range from the nearest quartile). N in the panel title denotes the total number of available data points.

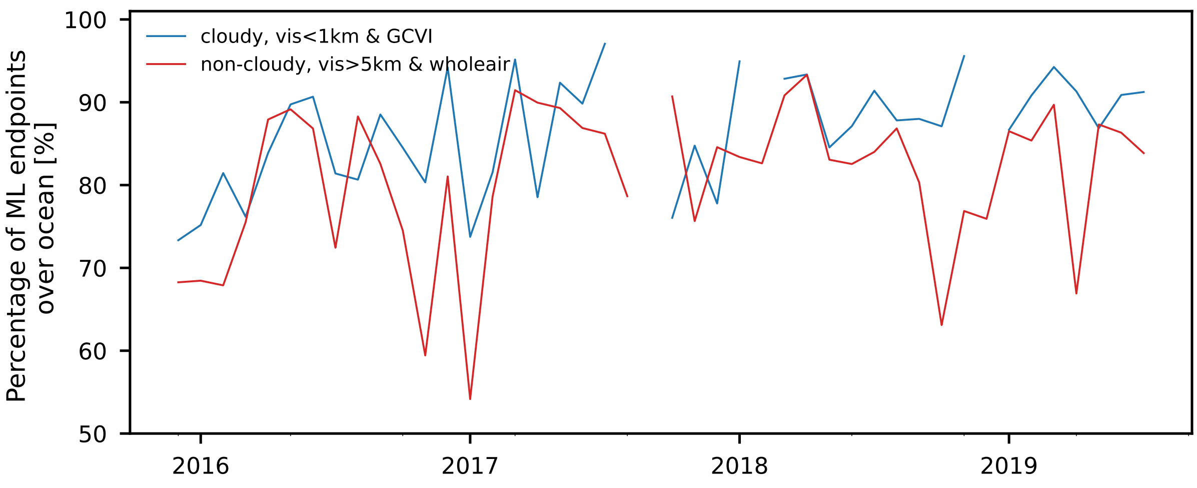

Figure S3. Percentage spent over the ocean of cloudy and cloud-free periods spent over each surface type (i.e. land or ocean) and within the mixed-layer (ML) is normalized by the total spent time within the ML. The average time spent over the ocean for cloudy periods equates to 86.85%, and 80.56% for cloud-free periods.

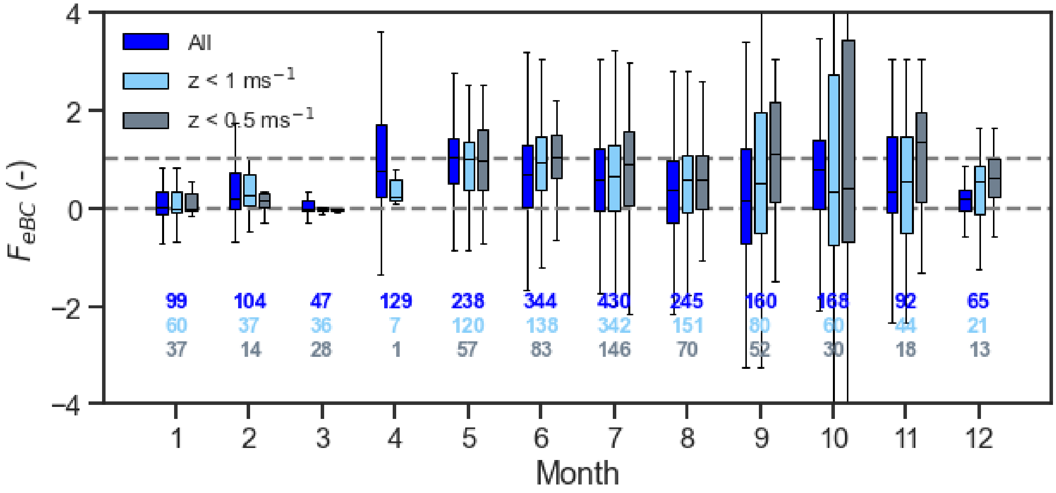

Figure S4. The annual cycle of the scavenged fraction of eBC. Scavenged fraction of eBC as a box plot for all data, and for vertical wind values below 1 ms^{-1} and 0.5 ms^{-1}, respectively. Shown are monthly mean and median values as solid and dashed line, respectively. The centre line of the boxes represents the median, while the extent of the boxes show the interquartile range. The whisker show the range of data (defined as 1.5 times the interquartile range from the nearest quartile). All box plots contain hourly mean values. The numbers in blue, light blue and grey give the number of hourly value contained in each month for each data class (see legend).

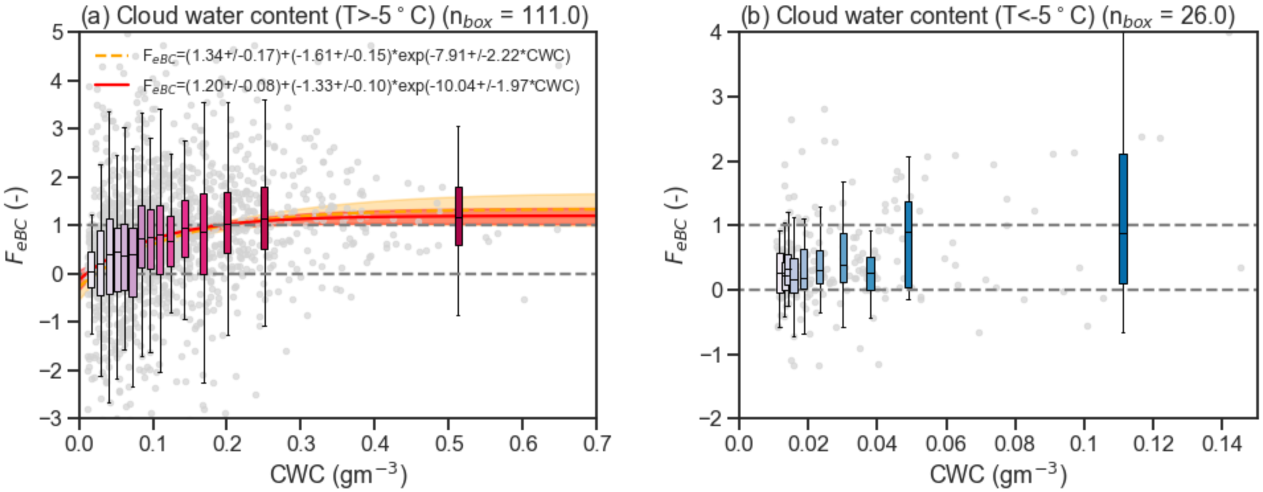

Figure S5. The scavenged fraction of eBC binned by cloud water content for two different temperature regimes. (a) Ambient temperature above -5°C and (b) for ambient temperature below -5°C. The number of points per box (15 in panel a and 10 in panel b) is given above each subplot. The centre line of the boxes represents the median, while the extent of the boxes show the interquartile range. The whisker show the range of data (defined as 1.5 times the interquartile range from the nearest quartile). The shading of the color of the box plots denotes the values of the x-axis. The exponential fits in panel a are shown for the 1-h mean values (orange dashed curve) and for the binned median values (red curve), respectively together with their corresponding 95 % confidence intervals (shaded area). The corresponding fit coefficients are given in the legend together with their 95 % confidence intervals. No reasonable exponential fit was possible for panel b.

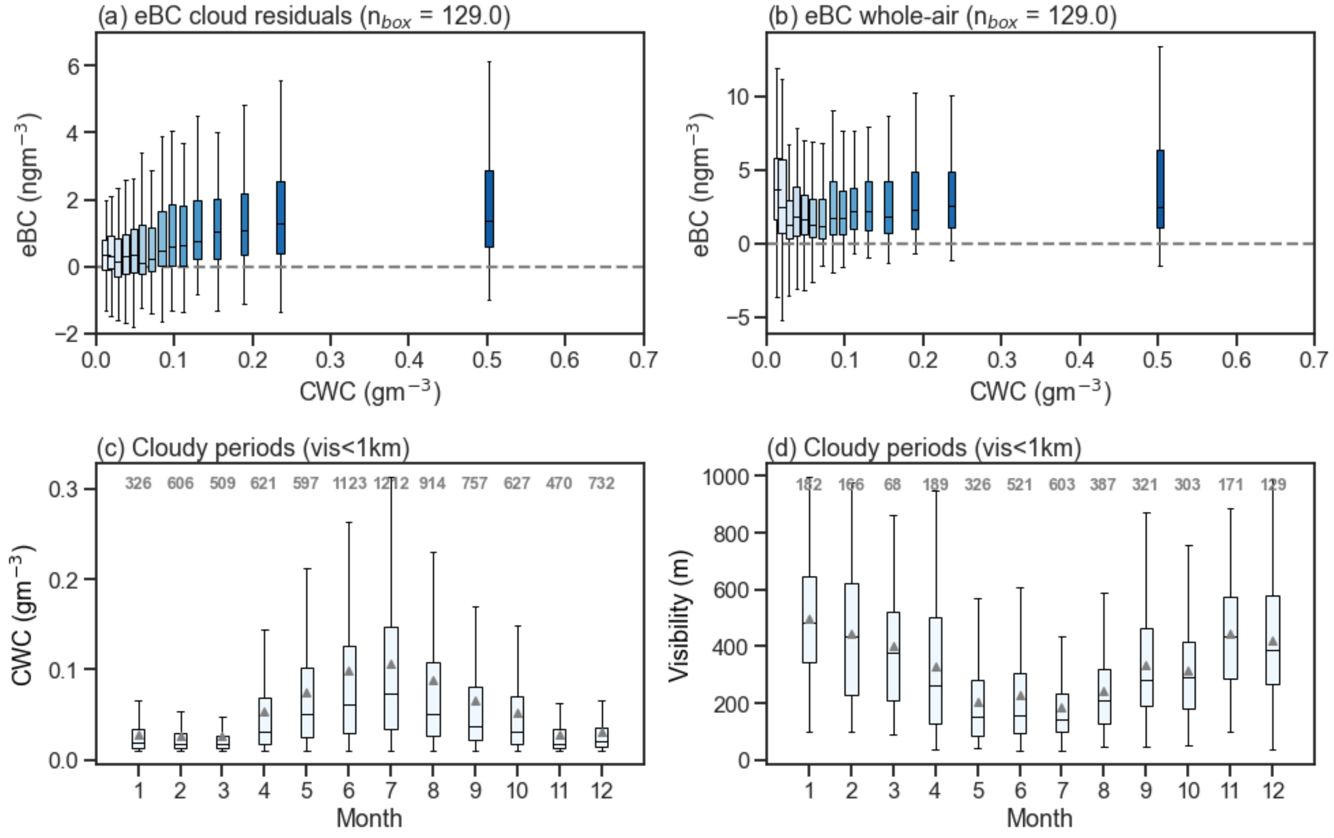

Figure S6. eBC concentrations binned by cloud water content (CWC) and annual cycle of CWC and visibility. (a) eBC within cloud residuals vs. CWC. (b) eBC of whole-air (during cloudy periods) vs. CWC. (c) Monthly values of CWC. (d) Corresponding monthly values of visibility. The centre line of the boxes represents the median, while the extent of the boxes show the interquartile range. The whisker show the range of data (defined as 1.5 times the interquartile range from the nearest quartile). The number of 1-h mean values in each box is given above the panel (a-b) or as grey number within the panel (c-d). The data in panel (a) and (b) is shown for all ambient temperatures.

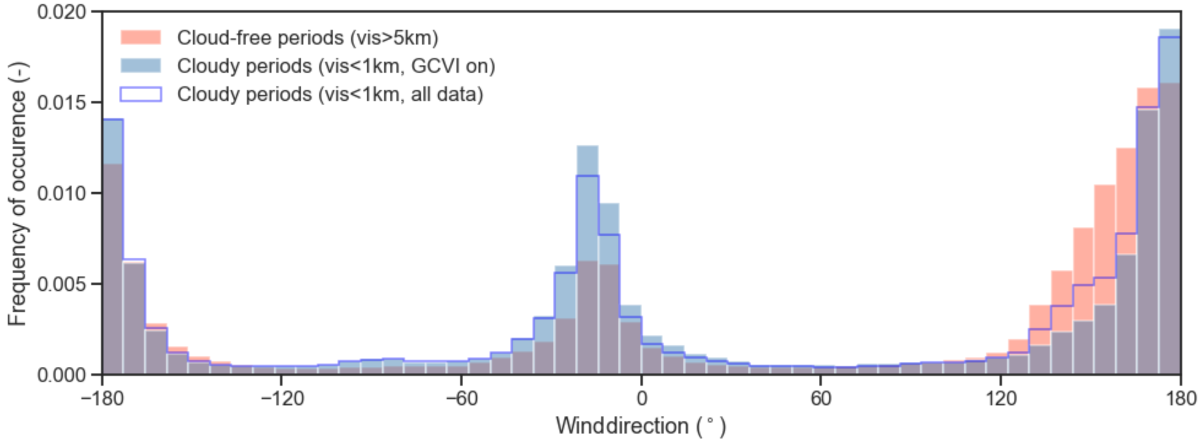

Figure S7. Normalised histogram of the occurrence of cloudy and cloud-free periods versus wind direction. Two wind directions prevail at Zeppelin Observatory: north-north-westerly winds which tend to bring more clouds, while south-south-easterly winds tend to bring less cloudy air. The difference between cloudy periods (visibility <1 km) with and without GCVI operation are shown with full and empty bars in blue.

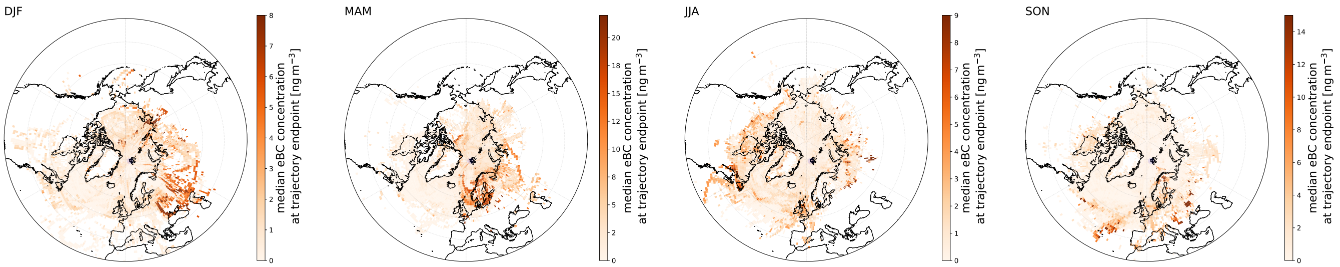

Figure S8. Source maps of eBC for cloudy periods for entire 4-year data set according to seasons. DFJ: winter, MAM: spring, JJA: summer, SON: autumn

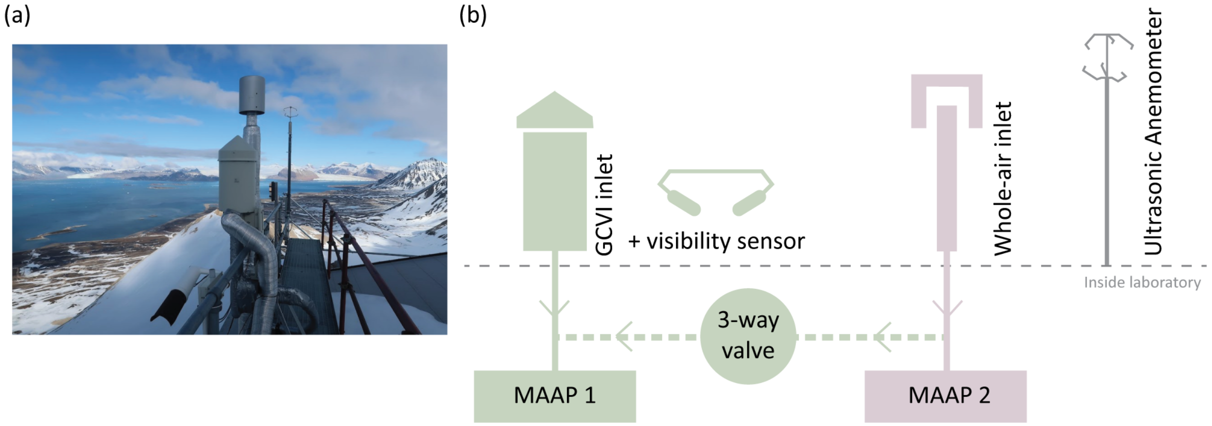

Figure S9. Set-up at Zeppelin Observatory, Ny-Ålesund, Svalbard. (a) View of the ground-based virtual impactor (GCVI) inlet (front), the whole-air inlet (behind GCVI) and the ultrasonic anemometer (in the background). (b) Schematic set-up. Two identical multi-angle absorption photometer (MAAP) instruments were sampling behind the GCVI (MAAP1) and whole-air (MAAP2) inlet. A 3-way valve enabled that MAAP1 sampled behind the whole-air inlet when the GCVI was turned off. The readings of a visibility sensor were used to determine the presence of a cloud at the observatory. An ultrasonic anemometer was used to record wind parameters.

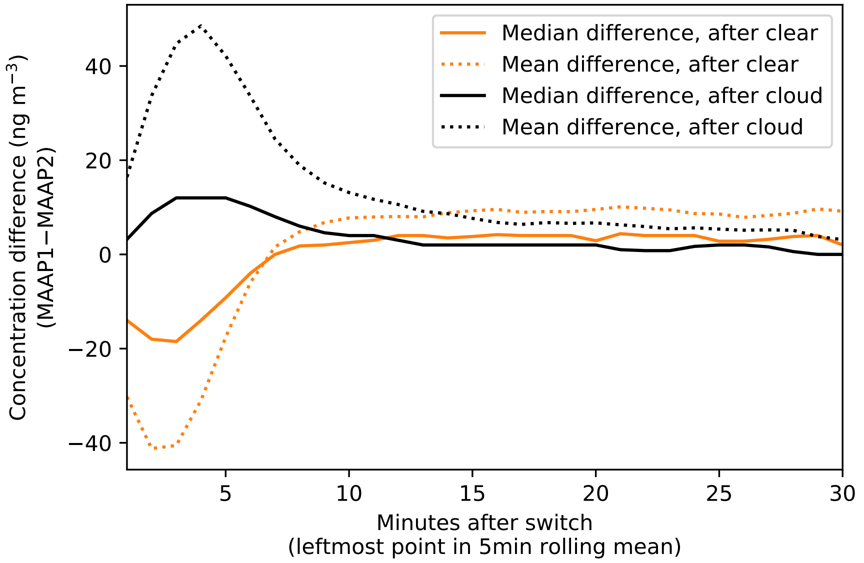

Figure S10. Concentration difference in eBC after the CVI was switched on (orange lines) or off (black lines). The switching of the GCVI caused fluctuations in the eBC readings due to humidity effects within the MAAP instruments. Values within 15 min after the switching of the GCVI were disregarded.

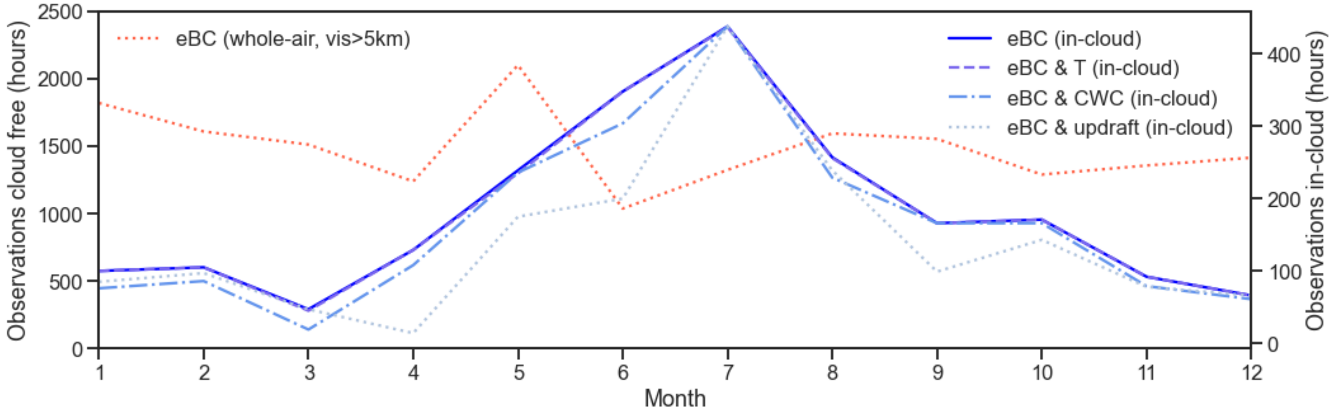

Figure S11. Available observations. Hours of observations during non-cloud periods (visibility>5 km, red dashed curve) and during cloudy periods when the GCVI was in operation (visibility<1 km). Shown are periods when both MAAP instruments measured eBC (blue solid line) and when corresponding auxiliary data was available for ambient temperature (blue dashed line), cloud water content (blue dashed dotted line) and updraft (blue dotted line).
